# Artificial intelligence computed tomography models for the discrimination of Wilms versus non-Wilms tumors: systematic review and meta-analysis

**DOI:** 10.1590/2175-8239-JBN-2025-0325en

**Published:** 2026-03-20

**Authors:** Burak Ardicli

**Affiliations:** 1 Hacettepe University Medical Faculty, Department of Pediatric Surgery, Ankara, Turkey.

To the Editor,

I read with great interest the article by Feitosa Filho et al. evaluating artificial intelligence (AI) models based on computed tomography for the differential diagnosis of Wilms and non-Wilms tumors^1^. The authors reported promising diagnostic performance, with a pooled specificity of approximately 83%, sensitivity of 64%, and an AUC of 0.831. This study addresses an increasingly relevant and technically sophisticated area of pediatric oncologic imaging, and I would like to raise several points for further discussion.

First, while the diagnostic performance metrics, including AUC, are compelling, the analysis does not clearly indicate whether potential confounders—such as patient age, tumor histology, bilateral disease, or prior treatment exposure—were considered. These variables can significantly influence both tumor appearance and radiomic features, potentially inflating the reported diagnostic accuracy. Notably, no multivariable adjustment for these clinical variables was performed in the meta-analysis, which may partially overestimate the model’s performance. Indeed, as acknowledged in the study’s own limitations, the lack of integration of clinical data into AI models further restricts generalizability and may contribute to the observed diagnostic accuracy. Future studies incorporating multivariable models or stratified analyses could yield more robust and clinically meaningful estimates of diagnostic accuracy in heterogeneous patient populations.

Second, the review synthesizes data from studies using heterogeneous imaging protocols, including differences in acquisition phases, slice thickness, and reconstruction algorithms. As acknowledged by the authors in the limitations section, this variability likely contributed to the substantial heterogeneity (I^2^) and wide confidence intervals observed across studies. Standardizing imaging acquisition and radiomic feature extraction protocols, or applying harmonization techniques such as ComBat while adhering to IBSI standards, would improve reproducibility and enhance the generalizability of AI models across centers.

Third, several included studies involved mixed histological findings, with varying proportions of non-Wilms tumors (e.g., CCSK, MRTK, RCC). As noted by the authors in the limitations section, grouping these heterogeneous subtypes into a single non-Wilms category may dilute or mask subtype-specific diagnostic challenges, given their distinct imaging characteristics. Performing subgroup or sensitivity analyses by histologic subtype would help clarify whether overall model performance is driven primarily by one or two dominant categories and improve interpretability for clinical application.

Fourth, the clinical applicability of AI models depends on their ability to guide clinical decision-making rather than solely improve diagnostic metrics. The present meta-analysis, while demonstrating promising diagnostic performance, does not address how these models could be integrated into real-world management algorithms. Linking AI outputs to clinical pathways—such as biopsy versus primary surgery or radical versus nephron-sparing surgery—will be essential to achieve real-world clinical impact and improve patient outcomes. In this regard, lessons learned from other oncologic domains, such as hepatology, where AI has already informed clinical workflows, may offer translatable frameworks^2^.

In conclusion, this meta-analysis makes a timely and valuable contribution to the growing evidence supporting the role of AI in the diagnostic characterization of pediatric renal tumors. However, translating these promising findings from technical validation to clinical implementation will require methodologically rigorous studies that integrate standardized imaging protocols, histology-specific analyses, and clearly defined clinical decision pathways. Addressing these elements will be essential to ensure reproducibility, improve patient selection, and enable evidence-informed surgical and oncologic strategies.
