# Supplementary material for: Artificial intelligence computed tomography models for the discrimination of Wilms versus non-Wilms tumors: systematic review and meta-analysis
Source: J Bras Nefrol. 2026 Mar 20;48(2):e20250325. doi: 10.1590/2175-8239-JBN-2025-0325en (PMC13004157; doi:10.1590/2175-8239-JBN-2025-0325en)
Supplement: Translation

# Translation: Modelos de tomografia computadorizada baseados em inteligência artificial para a distinção entre tumores de *Wilms* e não *Wilms*: revisão sistemática e meta-análise

**DOI:** 10.1590/2175-8239-JBN-2025-0325pt

**Authors:** Burak Ardicli

**Affiliations:** 1 Hacettepe University Medical Faculty, Department of Pediatric Surgery, Ankara, Turkey.

Ao Editor,

Li com grande interesse o artigo de Feitosa Filho et al., que avaliou modelos de inteligência artificial (IA) baseados em tomografia computadorizada para o diagnóstico diferencial de tumores de *Wilms* e não *Wilms* ^1^. Os autores relataram um desempenho diagnóstico promissor, com uma especificidade combinada de aproximadamente 83%, sensibilidade de 64% e uma AUC de 0,831. Este estudo aborda uma área cada vez mais relevante e tecnicamente sofisticada da imagem oncológica pediátrica. Gostaria de levantar alguns pontos para futuras discussões.

Primeiramente, embora as métricas de desempenho diagnóstico, incluindo AUC, sejam convincentes, a análise não indica claramente se potenciais fatores de confusão—como idade do paciente, histologia tumoral, doença bilateral ou exposição prévia a tratamento—foram considerados. Essas variáveis podem influenciar significativamente tanto a aparência do tumor quanto as características radiômicas, possivelmente inflacionando a precisão diagnóstica relatada. É importante notar que nenhum ajuste multivariável para essas variáveis clínicas foi realizado na meta-análise, o que pode superestimar parcialmente o desempenho do modelo. De fato, conforme reconhecido nas próprias limitações do estudo, a ausência de integração de dados clínicos aos modelos de IA restringe ainda mais a generalização dos resultados e pode contribuir para a precisão diagnóstica observada. Estudos futuros que incorporem modelos multivariáveis ou análises estratificadas podem gerar estimativas de precisão diagnóstica mais robustas e clinicamente significativas em populações heterogêneas de pacientes.

Em segundo lugar, a revisão sintetiza dados proveniente de estudos que utilizaram protocolos de imagem heterogêneos, incluindo diferenças nas fases de aquisição, espessura de corte e algoritmos de reconstrução. Conforme reconhecido pelos autores na seção de limitações, essa variabilidade provavelmente contribuiu para a heterogeneidade substancial (I^2^) e os amplos intervalos de confiança observados entre os estudos. A padronização dos protocolos de aquisição de imagens e extração de características radiômicas, ou a aplicação de técnicas de harmonização, como o ComBat, em conformidade com os padrões da *Imaging Biomarker Standardisation Initiative* (IBSI), contribuiria para aprimorar a reprodutibilidade e aumentar a generalização dos modelos de IA entre os centros.

Em terceiro lugar, diversos estudos incluídos envolveram achados histológicos mistos, com proporções variáveis de tumores não *Wilms* (por exemplo, CCSK, MRTK, RCC). Conforme observado pelos autores na seção de limitações, o agrupamento desses subtipos heterogêneos em uma única categoria de “não *Wilms*” pode diluir ou mascarar os desafios diagnósticos específicos de cada subtipo, dadas suas características distintas de imagem. A realização de análises de subgrupos ou de sensibilidade por subtipo histológico ajudaria a esclarecer se o desempenho global do modelo é impulsionado principalmente por uma ou duas categorias dominantes, além de melhorar a interpretabilidade para a aplicação clínica.

Quarto, a aplicabilidade clínica dos modelos de IA depende de sua capacidade de orientar a tomada de decisões clínicas, e não apenas de melhorar métricas diagnósticas. A presente meta-análise, embora demonstre um desempenho diagnóstico promissor, não aborda como esses modelos podem ser integrados a algoritmos de manejo do mundo real. Vincular os resultados da IA às vias clínicas—como biópsia versus cirurgia primária ou cirurgia radical versus cirurgia com preservação do néfron—será essencial para alcançar impacto clínico de mundo real e melhorar os desfechos dos pacientes. Nesse sentido, as lições aprendidas em outros domínios oncológicos, como a hepatologia, onde a IA já tem influenciado os fluxos de trabalho clínicos, podem oferecer modelos aplicáveis^2^.

Em conclusão, esta meta-análise traz uma contribuição oportuna e valiosa para o crescente corpo de evidências que sustenta o papel da IA na caracterização diagnóstica de tumores renais pediátricos. No entanto, para traduzir esses achados promissores da validação técnica para a implementação clínica, serão necessários estudos metodologicamente rigorosos que integrem protocolos de imagem padronizados, análises histológicas específicas e fluxos de decisão clínica claramente definidos. Abordar esses elementos será essencial para garantir a reprodutibilidade, aprimorar a seleção de pacientes e possibilitar estratégias cirúrgicas e oncológicas baseadas em evidências.
